# Copy number variation in exportin-4 (*XPO4*) gene and its association with histological severity of non-alcoholic fatty liver disease

**DOI:** 10.1038/srep13306

**Published:** 2015-08-21

**Authors:** Shamsul Mohd Zain, Zahurin Mohamed, Munir Pirmohamed, Hwa Li Tan, Mohammed Abdullah Alshawsh, Sanjiv Mahadeva, Wah-Kheong Chan, Nik Raihan Nik Mustapha, Rosmawati Mohamed

**Affiliations:** 1 The Pharmacogenomics Laboratory, Department of Pharmacology, Faculty of Medicine, University of Malaya, 50603 Kuala Lumpur, Malaysia; 2 Department of Pharmacology, Faculty of Medicine, University of Malaya, Kuala Lumpur, Malaysia; 3 The Wolfson Centre for Personalised Medicine, University of Liverpool, Liverpool, United Kingdom; 4 Department of Medicine, Faculty of Medicine, University of Malaya, Kuala Lumpur, Malaysia; 5 Department of Pathology, Hospital Sultanah Bahiyah, Alor Setar, Malaysia

## Abstract

A recent genome-wide copy number (CNV) scan identified a 13q12.11 duplication in the exportin-4 (*XPO4*) gene to be associated with non-alcoholic steatohepatitis (NASH). We sought to confirm the finding in a larger cohort and to assess the serum XPO4 pattern in a broad spectrum of non-alcoholic fatty liver disease (NAFLD) cases. We analysed 249 NAFLD patients and 232 matched controls using TaqMan assay and serum XPO4 was measured. Copy number distribution was as follows: copy number neutral (NAFLD: 53.8%, controls: 68.6%), copy number losses (NAFLD: 13.3%, controls: 12.9%), copy number gains (NAFLD: 32.9%, controls: 18.5%). CNV gain was significantly associated with a greater risk of NAFLD (adjusted OR 2.22, 95% CI 1.42–3.46, P = 0.0004) and NASH (adjusted OR 2.33, 95% CI 1.47–3.68, P = 0.0003). Interestingly, subjects carrying extra copy number showed significantly higher serum ALT and triglyceride (P < 0.05). Serum XPO4 levels progressively declined (P = 0.043) from controls (24.6 ng/mL) to simple steatosis (20.8 ng/mL) to NASH (13.8 ng/mL). In conclusion, *XPO4* CNV duplication was associated with histological severity of NAFLD, and accompanied by changes in serum XPO4 levels providing insights into NAFLD pathogenesis, and has the potential for biomarker development.

The recognition of single nucleotide polymorphisms (SNPs) as a major source of human genetic variation following completion of the Human Genome Project has been accompanied by rapid developments in high-throughput next generation sequencing (NGS) and a variety of genotyping platforms^1^. Availability of sophisticated technologies has greatly increased the resolution and depth of coverage across the entire genome to allow possible cataloguing of human genetic variation. The strategy of combining NGS and genome-wide association studies (NGS-GWAS) has been successful in identifying genetic markers (SNPs) associated with phenotypic variability and disease susceptibility, and has been applied to non-alcoholic fatty liver disease (NAFLD)^2^, a spectrum of disease ranging from essentially benign simple steatosis (fatty infiltration of the liver) to the more severe form non-alcoholic steatohepatitis (NASH— fat with inflammation and/or fibrosis); the latter can progress to cirrhosis and liver cancer^3^. But overall GWAS approaches have only identified a fraction of the heritability of NAFLD, and as of now, candidate gene approaches have been inconclusive in linking genetic variants to the severity of NAFLD with the exception of patatin-like phospholipase domain containing 3 (*PNPLA3*)^4^.

CNVs constitute a substantial fraction of genetic and phenotypic variability affecting segments greater than 1 kb in length—these include both duplications (copy number gains) and deletions (copy number losses) of genetic material^5^. CNVs have been estimated to affect ~12% of the human genome^6^ with over 1000 genes mapped within or close to CNV-affected regions^7^. Two genome-wide association scans have pioneered the study of CNV in NAFLD^8^,^9^. These have suggested that both the risk of non-alcoholic fatty liver disease (NAFLD) and its progression can be linked to DNA copy number variation (CNVs), which has added a whole new level of complexity to the study of molecular determinants of NAFLD.

We have identified several genomic regions that are potentially relevant to non-alcoholic steatohepatitis (NASH)^9^, a severe form of NAFLD, but these need to be confirmed in a larger set of samples. In the present study, we have further assessed CNVR 13q12.11 because (i) CNV gain at locus 13q is one of the highest differentially expressed CNVs in the hepatocellular carcinoma (HCC) genome^10^; (ii) it contains exportin-4 (*XPO4*), a tumor suppressor gene involved in the pathogenesis of HCC^11^; and (iii) a previous report has shown that *XPO4* is known to be expressed in both the liver and peripheral blood^12^. The *XPO4* gene is a member of the importin β family that mediates the nuclear-cytoplasmic transport of protein cargoes^13^. Zender and colleagues in their oncogenomics-based *in vivo* RNAi screen demonstrated that the reintroduction of *XPO4* selectively suppresses tumors including HCC with *XPO4* deletion^11^. Increased expression of *XPO* was correlated with better prognosis and survival rate among patients with HCC^14^,^15^. The expression of *XPO4* also seems to be significantly decreased in cirrhotic livers and after chronic hepatitis B infection when compared with normal healthy controls^12^.

## Results

### Study subjects

Subject demographic and clinical data are shown in Table 1. There were a total of 249 NAFLD patients: 110 (44%) were Malays, 86 (35%) Chinese and 53 (21%) Indians. Out of the 232 controls, 79 (34%) were Malays, 97 (42%) Chinese and 56 (24%) Indians. NAFLD patients and controls significantly differed (P < 0.05) in gender, BMI, HbA1c, liver enzymes and lipid profiles, but not in age. The differences in the parameters simply reflect the nature of the subjects according to the criteria set. NAFLD patients were further grouped into simple steatosis and NASH (Table 2). Significantly higher (P < 0.05) levels of histological parameters, waist circumference, BMI, HbA1c, and liver enzymes were observed in patients with NASH compared to those with simple steatosis.

### Association of CNV 13q12.11 with NAFLD

On the basis of the GWAS discovery, we followed up the CNVR on chromosome 13q12.11 in 481 case-control replication samples, in whom we detected eight subjects (1.7%) with homozygous deletions, 55 subjects (11.4%) with heterozygous deletions, 293 subjects (60.9%) with two copies, 99 subjects (20.6%) with three copies, 20 subjects (4.2%) with four copies, and six subjects (1.2%) with five copies. Overall, 134 NAFLD patients (53.8%) were copy number neutral, 33 (13.3%) had deletions and 82 (32.9%) had duplications. As for the controls, 159 (68.6%) were copy number neutral, 30 (12.9%) had copy number losses and 43 (18.5%) had copy number gains.

As shown in Table 3, the frequency of CNV gain was significantly higher in the NAFLD patients compared to the controls (adjusted OR 2.32, 95% CI 1.49–3.61, P = 0.0002). However, there was no significant difference between the two groups for CNV loss. CNV gain was also significantly associated with NASH (adjusted OR 2.43, 95% CI 1.54–3.83, P = 0.0001) but not with simple steatosis. When further collapsed into NASH with no significant fibrosis (fibrosis score <2) and NASH with significant fibrosis (fibrosis score ≥2), both groups were found to be significantly associated with CNV gain (adjusted OR 1.87, 95% CI 1.08–3.21, P = 0.029 and adjusted OR 3.21, 95% CI 1.90–5.72, P = 5.94 × 10^−5^, respectively) with a stronger effect observed in the latter group. We evaluated the association of CNV gain with NASH at the severe stage (fibrosis score ≥3; bridging fibrosis and cirrhosis) and found that patients with CNV gain possess 2.55 higher risk for advanced NASH (P = 0.012).

When the subjects were stratified by ethnicity, the frequency of CNV gain was found to be relatively high in the Malays (case: 43%, control: 27%), followed by the Chinese (case: 39%, control: 19%) and Indians (case: 27%, control: 18%). The CNV gain was associated with risk of NAFLD in the Malays (adjusted OR 2.02, 95% CI 1.02–3.99, P = 0.043) and Chinese (adjusted OR 2.80, 95% CI 1.32–5.95, P = 0.007) but not in the Indians. CNV gain was also associated with risk of NASH in the Chinese (adjusted OR 3.29, 95% CI 1.52–7.13, P = 0.003) (Supporting Information Table S1). Our replication study has a power of 98%, 22% and 98% with an α of 0.05 to detect associations with duplications at 13q12.11, for association with NAFLD, simple steatosis and NASH, respectively.

**Figure 1:**
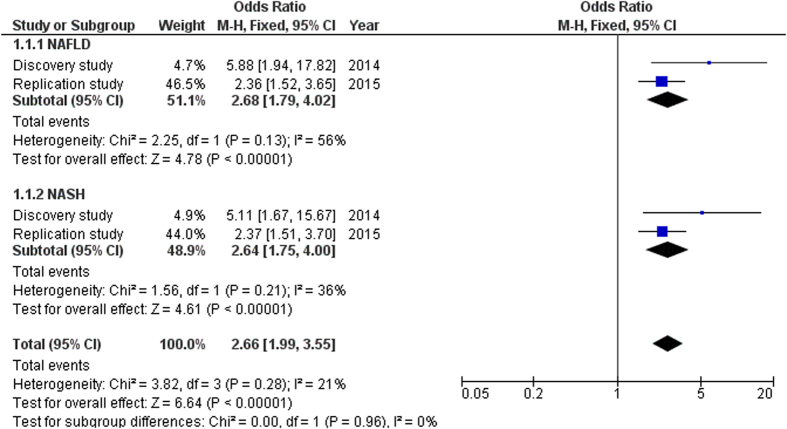
Association analysis between CNV gain and NAFLD. Estimations of odds ratios (OR) and 95% confidence intervals (CI) in each study are displayed as closed squares and horizontal lines, respectively. The size of the black squares reflects the weight of the study in the meta-analysis. The diamond represents the combined OR, calculated using a fixed effect model, with its 95% CI. *NAFLD* non-alcoholic fatty liver disease, *NASH* non-alcoholic steatohepatitis.

In order to further investigate the effect of the CNV gain, we first validated the 67 (68%) available samples from the 98 discovery samples^9^ using qPCR. The frequency of CNV gain (20.7%) in the control discovery samples was relatively similar to that of the controls from the replication samples (18.5). Results from the discovery samples indicated that CNV gain at 13q12.11 was associated with risk of NAFLD (OR 5.88, 95% CI 1.94–17.82, P = 0.002) and NASH (OR 5.11, 95% CI 1.67–15.67, P = 0.004), but not with simple steatosis. Then, we performed a meta-analysis including both discovery and replication studies (Fig. 1), which confirmed the significant association with NAFLD (OR 2.68, 95% CI 1.79–4.02, P < 0.0001) and NASH (OR 2.64, 95% CI 1.75–4.00, P < 0.0001).

### Comparison of clinical parameters by different copy number status

We compared clinical parameters in NAFLD patients with different copy number status (Table 4). Significant differences in the serum were found with ALT (P = 0.038), GGT (P = 0.007) and triglycerides levels (P = 0.046); both serum ALT and triglyceride are indicators for NAFLD severity. Higher copy numbers were associated with higher serum ALT (copy number deletion vs. neutral vs. duplication: 72.1 vs. 82.3 vs. 90.3 IU/L) and triglycerides (136.0 vs. 154.1 vs 160.9 mg/dL). However, after adjustments for the covariates such as age, gender and ethnicity, the differences were no longer significant (Table 4). To investigate the magnitude and direction of the effect, using linear regression, we revealed positive correlations between CNV gains and log-transformed serum ALT (P = 3.20 × 10^−5^) and triglycerides (P = 0.004). Spearman’s correlation also showed a similar trend (P = 4.01 × 10^−5^ and P = 0.003, respectively).

### Sensitivity analysis

Because triglyceride is a NAFLD-associated risk factor^16^, and there was a significant correlation between the CNV and serum triglyceride, we wanted to exclude the possibility of collider stratification bias. We therefore performed sensitivity analysis by excluding those patients with serum triglyceride levels that were above normal. The associations remained unchanged for NAFLD (OR 2.10, 95% CI 1.27–3.48, P = 0.004), NASH (OR 2.23, 95% CI 1.32–3.75, P = 0.003), and NASH with significant fibrosis (OR 3.02, 95% CI 1.61–5.69, P = 0.001) while simple steatosis was still not significantly associated. NASH with non-significant fibrosis lost significance. This again suggests that the CNV gain at locus 13q12.11 is associated with more severe degrees of NASH.

### Serum XPO4 levels and indices of liver damage

Serum XPO4 levels were shown to be significantly lower in NAFLD patients (16.3 ng/mL) compared to the controls (24.6 ng/mL) (P = 0.038; Table 1). There was a reduction in XPO4 levels going from controls (24.6 ng/mL) to simple steatosis (20.8 ng/mL) and NASH (13.8 ng/mL) (P = 0.043; Table 2). As expected, the serum XPO4 levels were not different between controls and simple steatosis but were significantly different between controls and NASH (P = 0.014). We then assessed the effect of CNV 13q12.11 on serum XPO4 levels: there was a suggestion of a decrease in levels with extra copies of the DNA segment, although this was not statistically significant (CNV losses– 23.0 ng/mL; CNV neutral– 20.4 ng/mL; and CNV gains– 18.0 ng/mL; Table 4).

## Discussion

In this study, using a larger cohort of NAFLD patients with well-defined histological characteristics, we extended our previous work^9^ and confirmed the association between the CNV 13q12.11 in the *XPO4* gene and NAFLD. The *XPO4* gene is a tumour suppressor gene located on the long arm of chromosome 13. Previous studies have suggested that *XPO4* plays a role in the initiation of HCC as its expression level decreases with the development of cancer^14^,^15^. Furthermore, the inactivation of *XPO4* is associated with HCC development in mice^11^. In the current study, we have shown that CNV gains in *XPO4* are associated with severity of NAFLD, in particular an association between the CNV and NASH with significant fibrosis. Lower serum XPO4 levels were found in patients with NAFLD when compared to healthy controls. Thus, we suggest that the presence of CNV in the *XPO4* gene is a predictor of the histological severity of NAFLD. Taken together, our data suggest that *XPO4* CNV duplication is associated with histological severity of NAFLD especially with that of NASH, but may not be a distinctive marker for the progression from simple steatosis to NASH.

In our CNV association study, we demonstrated that CNV gains may also be associated with increased serum ALT and triglyceride levels. Although it is unclear how the variations may cause these abnormalities, we speculate that it is related to the involvement of *XPO4* in the signal transduction and nuclear export of SMAD family member 3 (Smad3) protein. Smad3 is an intracellular mediator of transforming growth factor beta 1 (TGF-β1) that has a multifaceted regulatory effect on metabolic homeostasis^17^. The downstream effects of Smad3 were observed in the aggravation of insulin resistance and adiposity^18^,^19^. By contrast, *Smad3*-knockout mice exhibited improved insulin sensitivity and β-oxidation thereby ameliorating glucotoxicity and lipotoxicity in several organs including the liver^18^. However, the effects of the CNV on these parameters need to be confirmed in other populations and using larger samples.

The *XPO4* gene is down-regulated in HCC tissues compared with normal tissue^14^,^15^. Recently, Zhang and colleagues showed that the expression was also lower in HCC when compared to liver cirrhosis and chronic hepatitis B^12^. In this study, we also report for the first time that serum XPO4 levels decrease in NAFLD with an association with disease severity. This trend may be due to the role of *XPO4* as a tumor suppressor. XPO4 mediates TGF-β1 that recruits and phosphorylates Smad3, and the resulting phosphorylation causes Smad3 to bind to DNA to modulate transcriptional events^20^. Notably, proteins such as XPO4 which are associated with signal transduction, have been found to be lowly expressed as the degree of phosphorylation increases^21^. Our data also suggest that serum XPO4 levels may decline in relationship to the number of copies, although this was not significant. This potential functional effect needs further study, particularly since there seems to be a trend to a decrease in levels as the copy number increases. Interestingly, although most CNVs are gene dosage insensitive^22^, about 10% of the CNV duplications in the human genome are dosage reversed^23^,^24^. This may be due to reduced transcription and gene silencing as a consequence of the gene duplication^22^.

Our study has several limitations. Among them is the relatively small number of simple steatosis patients which may have resulted in the negative association in this sub-group. However, since the observed association was consistent with the discovery study^9^, we feel the association is likely to hold true. In addition, Royo *et al*. in a genome-wide scan in a set of simple steatosis patients did not find an association of CNV 13q12.11 with simple steatosis^8^. This limitation is also due to the fact that the recruitment centre, UMMC is a tertiary referral center where more severely affected patients, i.e. those with NASH, are likely to be seen. Association comparison analysis was not performed in homozygous deletion, subjects with four copies and subjects with five copies due to limitation in the sample size. It is also important to note that in the discovery study using array comparative genomic hybridization (aCGH), this CNV was exclusively found only in NASH but not in controls^9^. However, the present study showed that the frequency of CNV gain was doubled in NASH (33.6%) compared to controls (18.5%). The observed events could be due to the following reasons: (i) The discovery sample in the genome-wide study was relatively low, (ii) in aCGH from the discovery study, the signal ratio between a case and control sample is normalised and converted to a log2 ratio, which is used for copy number call. Detection in the control may have been seen but when the ratio does not reach significant level, copy number is not called, and (iii) unlike monogenic disease with high penetrance and clear patterns of inheritance, NAFLD is a polygenic disease with greatly varying degrees of penetrance. Validation studies in a prospective setting are warranted. Our data were also not supported by *XPO4* gene expression levels which would have added strength to the findings. The strength of this study, however, is that the association findings were consistent with the discovery GWAS^9^. Furthermore, meta-analysis of the data from the discovery and replication studies confirmed the association with NASH (Fig. 1). To the best of our knowledge, this is the first study to confirm the previous genome-wide report on the association of the CNV with NASH.

In conclusion, we have demonstrated and confirmed the association of CNV gains at locus 13q12.11 with NASH. Lower serum XPO4 levels were observed in patients with NASH compared to those with simple steatosis and there was a suggestive decline in levels of serum XPO4 with extra copy number. Knowledge of the biological actions of *XPO4* will enhance our understanding of its role in NAFLD progression. This study also needs to be replicated in a larger cohort and in various other ethnic populations.

## Methods

### Study subjects

We included 249 consecutive biopsy-proven NAFLD patients from the University Malaya Medical Centre (UMMC). NAFLD patients were confirmed through liver histology upon detection of symptoms or signs that are attributable to liver disease through imaging, or upon discovery of abnormal liver biochemistry^25^. NAFLD stages were evaluated according to the NASH Clinical Research Network (CRN) criteria^26^,^27^. All liver biopsy specimens were on average 1.5 cm long and contained at least six portal tracts. There was no evidence of Hepatitis B nor Hepatitis C infection, autoimmune hepatitis, history of alcohol consumption >10g/day^28^, exposure to drugs known to cause steatosis or Wilson’s disease reported in any subjects. Based on the NASH CRN, NAFLD patients were grouped into simple steatosis (n = 32) and NASH (n = 217), the latter was further stratified into NASH without significant fibrosis (fibrosis score <2, n = 114) and NASH with significant fibrosis (fibrosis score ≥2, n = 103)^29^. For subsequent analysis, NASH was also grouped into early NASH (fibrosis score ≤2, n = 167) and advanced NASH (fibrosis score ≥3, n = 50)^25^.

All controls (n = 232) were genetically unrelated healthy subjects, confirmed to have normal liver function and had no indication of fatty liver as determined by the following parameters: body mass index (BMI) <23 kg/m^2^, fasting plasma glucose <110 mg/dL, and normal lipid profile. NAFLD was actively excluded in the controls by ultrasonography according to the absence of the following criteria: (i) slight diffuse increase in bright homogeneous echoes in the liver parenchyma with normal visualization of the diaphragm and portal and hepatic vein borders, and normal hepatorenal echogenicity contrast; (ii) diffuse increase in bright echoes in the liver parenchyma with slightly impaired visualization of the peripheral portal and hepatic vein borders; and (iii) marked increase in bright echoes at a shallow depth with deep attenuation, impaired visualization of the diaphragm and marked vascular blurring^30^. All experimental protocols were approved by the responsible Medical Ethic Committee of UMMC (ethics reference number: 702.11) and the methods were carried out in accordance with the approved guidelines. Written informed consent was obtained from all the patients prior to recruitment into the study.

### Biochemical and clinical assessments

Anthropometric data such as height and weight for the determination of body mass index (BMI, kg/m^2^), and waist circumference, were determined using standard protocols. Measurement of blood pressure (mmHg) was according to standard recommendation and clinical practice guidelines. The biochemical tests for the determination of hemoglobin A1c (HbA1c), high-density lipoprotein cholesterol (HDL), low-density lipoprotein cholesterol (LDL), total cholesterol, triglycerides, alanine transferase (ALT), aspartate aminotransferase (AST), and gamma-glutamyl transpeptidase (GGT) levels were according to standard clinical laboratory methods carried out in an accredited laboratory at UMMC.

### Measurement of serum XPO4 levels

From the serum samples available, we randomly selected subjects that represented different copy number status from different disease stage (42 controls– 7 losses, 23 neutral and 12 gains; 19 simple steatosis– 2 losses, 9 neutral and 8 gains; and 34 NASH– 5 losses, 18 neutral and 11 gains). XPO4 evaluation was performed on an aliquot of serum collected after overnight fasting at the time of sampling and stored at −80 ^°^C. Serum XPO4 levels were determined using a commercial enzyme-linked immunosorbent assay (ELISA) kit (SunRed Biotech, Shanghai, PRC) according to the manufacturer’s recommendations, the lowest limit of detection being 0.172 ng/mL.

### CNVR 13q12.11 genotyping

Genomic DNA was extracted from the blood samples using the QiAamp DNA Mini Kit (Qiagen, Hilden, Germany). The extracted DNA with good quality (OD_260_/OD_280_ = 1.8–2.0) was diluted to a final concentration of 5 ng/μL. The Applied Biosystems protocols that use a duplex TaqMan real-time quantitative polymerase chain reaction (qPCR) method were employed to call for CNV (13q12.11: Assay Hs03857719_cn) for every sample. Basically, each reaction (20 μL) contained 10 μL master mix, 1 μL TaqMan Copy Number Assay, 1 μL TaqMan Copy Number Reference Assay, 4 μL nuclease free water, and 4 μL genomic DNA. All reactions were run in quadruplicate with PCR cycling conditions as follows: 1 PCR cycle at 95 °C for 10 min, followed by 40 cycles at 95 °C for 15 sec and 60 °C for 1 min. Negative controls were introduced for every run to ensure the genotyping quality.

### CNV validation and meta-analysis

Validation of the previous CNV typing was done on the available samples using qPCR as above. To add strength to the findings, results of the discovery study and replication study were meta-analysed.

### Statistical analysis

All statistical tests were performed using SPSS version 16.0 (IBM Corp., Chicago, IL, USA), unless otherwise mentioned. Data were presented as percentage or mean ± standard deviation (S.D). Categorical and continuous variables were compared between NAFLD patients and controls using Pearson’s χ^2^ test, independent t-test and Mann-Whitney U test as appropriate. Odds ratios and 95% confidence interval (CI) for the findings were computed using logistic regression. Multivariate analysis revealed that gender was a contributing factor for NAFLD, and hence, gender was adjusted in the subsequent analysis. We also included age in the adjustment despite the fact that it was matched between NAFLD patients and controls as age is a known risk factor for NAFLD. Parameter comparisons among CNV status were tested using Analysis of Variance (ANOVA) and Kruskal-Wallis as appropriate. Subsequently, Analysis of Covariance (ANCOVA) using the general linear model was applied with age, gender and ethnicity as covariates. The P-values were corrected for multiple testing using the false discovery rate (FDR) method from the Benjamini-Hochberg procedure^31^. Linear regression was used to assess the correlation between genetic variants and clinical parameters for normally distributed variables; otherwise Spearman’s correlation test was adopted. Variables were log-transformed to achieve normality. Sensitivity analysis was performed according to the approach by Mefford and Witte^32^. A two-sided P-value of <0.05 is considered to be statistically significant.

Meta-analysis was conducted using the Review Manager (RevMan 5.3) of the Cochrane Collaboration utilising a Mantel-Haenszel test to estimate the pooled ORs and corresponding 95% CIs by assuming either fixed or random effect meta-analysis, where appropriate.

Power calculations were performed using Quanto power calculator version 1.2.4, with the following assumptions: the CNV frequency was 0.19, the baseline risk for the Malaysian population was 0.17^33^,^34^ and the detectable odds ratio ranged from 1.5–2.0.

## Additional Information

**How to cite this article**: Zain, S. M. *et al*. Copy number variation in exportin-4 (*XPO4*) gene and its association with histological severity of non-alcoholic fatty liver disease. *Sci. Rep*. **5**, 13306; doi: 10.1038/srep13306 (2015).

## Supplementary Material

Supplementary Information

## Figures and Tables

**Table 1:** Demographic and clinical data of the subjects.

Characteristics	*n* (%) or Mean ± SD		p-value*	p-value**
	Control (*n* = 232)	NAFLD (*n* = 249)		
Gender			0.043	—
Males	96 (41)	126 (51)	—	—
Females	136 (59)	123 (49)	—	—
Ethnicity			0.073	—
Malay	79 (34)	110 (44)	—	—
Chinese	97 (42)	86 (35)	—	—
Indian	56 (24)	53 (21)	—	—
Age (years)	49.6 ± 13.1	50.6 ± 11.6	0.307	0.102
BMI (kg/m^2^)	21.6 ± 1.7	29.2 ± 4.5	<0.0001	<0.0001
HbA1c (%)	5.3 ± 0.4	6.5 ± 1.6	<0.0001	<0.0001
HDL cholesterol (mg/dl)	49.3 ± 11.9	46.9 ± 11.5	0.30	0.014
LDL cholesterol (mg/dl)	83.2 ± 17.2	116.9 ± 41.0	<0.0001	<0.0001
Total cholesterol (mg/dl)	174.0 ± 34.0	195.0 ± 44.0	<0.0001	<0.0001
Triglycerides (mg/dl)	105.6 ± 36.5	154.8 ± 68.7	<0.0001	<0.0001
AST (IU/L)	20.3 ± 5.1	46.7 ± 27.1	<0.0001	<0.0001
ALT (IU/L)	24.4 ± 6.2	83.0 ± 48.2	<0.0001	<0.0001
GGT (IU/L)	36.5 ± 17.3	106.4 ± 103.0	<0.0001	<0.0001
Serum XPO4 (ng/mL)^a^	24.6 ± 18.5	16.3 ± 14.8	0.038	0.048
*ALT* alanine transferase, *AST* aspartate aminotransferase, *BMI* body mass index, *GGT* gamma glutamyl transpeptidase, *HbA1c* haemoglobin A1c, *HDL* high-density lipoprotein, *LDL* low-density lipoprotein, *NAFLD* non-alcoholic fatty liver disease, *XPO4* exportin-4.				

^a^Number of subjects (n) was not equal to overall subjects.

^*^P-values obtained using Mann-Whitney U test except for gender and ethnicity used χ^2^ test.

^**^P-values obtained using ANCOVA with age, gender and ethnicity as covariates.

**Table 2:** Demographic and clinical data of the NAFLD patients.

Characteristics	n (%) or Mean ± SD		
	Simple steatosis (n = 32)	NASH (n = 217)	p-value
Gender, n (%)			0.461
Males	18 (56)	108 (50)	
Females	14 (44)	109 (50)	
Age (years)*	50.4 ± 10.4	50.7 ± 11.8	0.906
BMI (kg/m^2^)*	27.4 ± 4.4	29.5 ± 4.4	0.012
HbA1c (%)	6.1 ± 1.5	6.6 ± 1.5	0.010
Waist circumference (cm)^a^	92.6 ± 11.4	96.5 ± 10.8	0.047
HDL cholesterol (mg/dl)*	47.0 ± 14.6	46.8 ± 11.0	0.942
LDL cholesterol (mg/dl)*	113.4 ± 43.1	117.4 ± 40.7	0.594
Total cholesterol (mg/dl)*	196.3 ± 40.7	194.8 ± 44.6	0.854
Triglycerides (mg/dl)	155.6 ± 86.2	153.5 ± 66.0	0.802
AST (IU/L)	33.4 ± 17.0	48.7 ± 27.8	0.001
ALT (IU/L)	60.7 ± 39.8	86.4 ± 48.6	0.001
GGT (IU/L)	101.1 ± 128.3	107.2 ± 99.0	0.019
Systolic blood pressure (mmHg)	132.7 ± 13.6	137.6 ± 18.7	0.144
Diastolic blood pressure (mmHg)	84.9 ± 11.5	85.7 ± 11.8	0.756
Serum XPO4 (ng/mL)^a^	20.8 ± 19.9	13.8 ± 10.2	0.141
*ALT* alanine transferase, *AST* aspartate aminotransferase, *BMI* body mass index, *GGT* gamma glutamyl transpeptidase, *HbA1c* haemoglobin A1c, *HDL* high-density lipoprotein, *LDL* low-density lipoprotein, *NAFLD* non-alcoholic fatty liver disease, *NASH* non-alcoholic steatohepatitis, *XPO4* exportin-4.			

^*^Number of subjects (n) was not equal to overall subjects.

^a^P-values obtained using independent t-test, all other comparisons used Mann-Whitney U test.

**Table 3:** Association tests of CNV gain with different NAFLD stages.

NAFLD spectrum	CNV gain frequency	Unadjusted		Adjusted for age, gender and ethnicity	
		p-value	OR (CI)	p-value	OR (CI)
*Control as reference*					
NAFLD vs. control	0.33 vs. 0.19	0.0002	2.26 (1.47-3.50)	0.0002	2.32 (1.49-3.61)
Simple steatosis vs. control	0.28 vs. 0.19	0.244	1.66 (0.71-3.92)	0.240	1.69 (0.70-4.08)
All NASH vs. control	0.34 vs. 0.19	0.0002	2.37 (1.51-3.70)	0.0001	2.43 (1.54-3.83)
NASH^a^ vs. control	0.31 vs. 0.19	0.013	1.96 (1.15-3.33)	0.029	1.87 (1.08-3.21)
NASH^b^ vs. control	0.37 vs. 0.19	0.0001	2.93 (1.70-5.04)	<0.0001	3.21 (1.90-5.72)
NASH^c^ vs. control	0.34 vs. 0.19	0.0003	2.41 (1.50-3.88)	0.0016	2.22 (1.35-3.63)
NASH^d^ vs. control	0.34 vs. 0.19	0.022	2.25 (1.13-4.48)	0.012	2.55 (1.27-5.21)
*Simple steatosis as reference*					
All NASH vs. simple steatosis	0.34 vs. 0.28	0.410	1.42 (0.61-3.30)	0.590	1.27 (0.53-3.04)*
NASH^a^ vs. simple steatosis	0.31 vs. 0.28	0.717	1.18 (0.49-2.86)	0.984	0.98 (0.38-2.51)*
NASH^b^ vs. simple steatosis	0.37 vs. 0.28	0.216	1.76 (0.72-4.30)	0.288	1.68 (0.66-4.20)*
NASH^c^ vs. simple steatosis	0.34 vs. 0.28	0.397	1.45 (0.62-3.41)	0.650	1.25 (0.54-2.96)*
NASH^d^ vs. simple steatosis	0.34 vs. 0.28	0.554	1.35 (0.50-3.64)	0.901	1.06 (0.36-3.21)*
*NASH*^a^ *as reference*					
NASH^b^ vs NASH^a^	0.37 vs 0.31	0.184	1.49 (0.83-2.70)		
*NASH*^c^ *as reference*					
NASH^d^ vs NASH^c^	0.34 vs 0.34	0.842	0.93 (0.47-1.86)		
*CI* confident interval, *OR* odds ratio, *NAFLD* non-alcoholic fatty liver disease, *NASH* non-alcoholic steatohepatitis, *NASH*.					

^*^P-values additionally adjusted for BMI, waist circumference and HbA1c.

*NASH^a^* non-alcoholic steatohepatitis without significant fibrosis

*NASH^b^* non-alcoholic steatohepatitis with significant fibrosis

*NASH^c^* early non-alcoholic steatohepatitis

*NASH^d^* advanced non-alcoholic steatohepatitis.

**Table 4:** Comparison of various clinical and histological parameters between the CNV status among NAFLD patients.

Characteristics	CNV status, n = 249 (Mean ± SD)				p-value**
	Losses (n = 33)	Neutral (n = 134)	Gains (n = 82)	p-value*	
Age (years)^a^	50.2 ± 11.3	51.1 ± 11.7	50.2 ± 12.0	0.835	0.961
BMI (kg/m^2^)^a^	28.3 ± 4.4	29.0 ± 4.8	29.8 ± 3.7	0.235	0.249
HbA1c (%)	6.4 ± 1.3	6.5 ± 1.5	6.5 ± 1.7	0.875	0.947
Waist circumference (cm)^a^	93.5 ± 10.2	96.2 ± 12.1	96.3 ± 9.1	0.404	0.393
HDL cholesterol (mg/dl)^a^	49.4 ± 9.2	46.2 ± 11.6	47.3 ± 10.9	0.316	0.345
LDL cholesterol (mg/dl)^a^	113.6 ± 36.9	115.3 ± 38.0	119.2 ± 44.1	0.697	0.692
Total cholesterol (mg/dl)^a^	185.4 ± 49.6	193.4 ± 41.9	200.1 ± 45.7	0.250	0.238
Triglycerides (mg/dl)	136.0 ± 58.2	154.1 ± 69.6	160.9 ± 66.8	0.046	0.190
AST (IU/L)	40.3 ± 25.7	46.2 ± 26.4	50.8 ± 27.7	0.063	0.167
ALT (IU/L)	72.1 ± 43.4	82.3 ± 52.0	90.3 ± 44.6	0.038	0.179
GGT (IU/L)	88.1 ± 110.4	102.3 ± 102.3	125.9 ± 106.2	0.007	0.206
Systolic BP (mmHg)	134.3 ± 18.8	137.8 ± 19.1	136.4 ± 16.7	0.732	0.574
Diastolic BP (mmHg)	85.3 ± 10.2	85.7 ± 10.9	85.5 ± 13.9	0.844	0.946
Steatosis grade	1.7 ± 0.7	1.9 ± 0.7	2.0 ± 0.7	0.105	0.115
Lobular inflammation	1.4 ± 0.5	1.4 ± 0.6	1.4 ± 0.6	0.922	0.932
Ballooning	1.2 ± 0.6	1.1 ± 0.7	1.2 ± 0.6	0.490	0.439
Fibrosis	1.7 ± 1.0	1.3 ± 1.1	1.6 ± 1.1	0.127	0.107
Serum XPO4 (ng/mL)	23.0 ± 16.1	20.4 ± 18.0	18.0 ± 16.3	0.304	0.663
*ALT* alanine transferase, *AST* aspartate aminotransferase, *BMI* body mass index, *GGT* gamma glutamyl transpeptidase, *HbA1c* haemoglobin A1c, *HDL* high-density lipoprotein, *LDL* low-density lipoprotein, *NAFLD* non-alcoholic fatty liver disease.					

^*^P-values

^**^P-values obtained using ANCOVA with age, gender and ethnicity as covariates.

^a^obtained using ANOVA, while Kruskal-Wallis test used for the other comparisons.
