# Supplementary material for: Copy number variation in exportin-4 (XPO4) gene and its association with histological severity of non-alcoholic fatty liver disease
Source: Sci Rep. 2015 Aug 21;5:13306. doi: 10.1038/srep13306 (PMC4543956; doi:10.1038/srep13306)
Supplement: Supplementary Information [file srep13306-s1.pdf]

Copy number variation in exportin-4 (*XPO4*) gene and its association with histological severity of non-alcoholic fatty liver disease

Shamsul Mohd Zain[1,2], Zahurin Mohamed[1,2], Munir Pirmohamed[3], Hwa Li Tan[1,2], Mohammed Abdullah Alshawsh[2], Sanjiv Mahadeva[4], Wah-Kheong Chan[4], Nik Raihan Nik Mustapha[5], Rosmawati Mohamed[4]

[1]The Pharmacogenomics Laboratory, [2]Department of Pharmacology, Faculty of Medicine, University of Malaya, Kuala Lumpur, Malaysia, [3]The Wolfson Centre for Personalised Medicine, University of Liverpool, Liverpool, United Kingdom, [4]Department of Medicine, Faculty of Medicine, University of Malaya, Kuala Lumpur, Malaysia, [5]Department of Pathology, Hospital Sultanah Bahiyah, Alor Setar, Malaysia

[*]Corresponding author: The Pharmacogenomics Laboratory, Department of Pharmacology, Faculty of Medicine, University of Malaya, 50603 Kuala Lumpur, Malaysia. E-mail: shamsulmohdzain@gmail.com, tanhwali@gmail.com

Table S1 Association tests of CNV gain with different NAFLD stages according to ethnicity

NAFLD spectrum	Malay		Chinese		Indian	
	p-value	OR (CI)	p-value	OR (CI)	p-value	OR (CI)
NAFLD vs. control	0.043	2.02 (1.02-3.99)	0.007	2.80 (1.32-5.95)	0.259	1.75 (0.66-4.65)
Simple steatosis vs. control	0.125	2.77 (0.75-10.22)	0.669	0.62 (0.07-5.45)	0.665	1.55 (0.21-11.20)
NASH vs. control	0.062	1.95 (0.97-3.94)	0.003	3.29 (1.52-7.13)	0.263	1.78 (0.65-4.86)
NASH vs. simple steatosis	0.473	0.60 (0.15-2.42)*	0.164	5.16 (0.51-52.10)*	0.722	1.41 (0.21-9.57)*

*P-values additionally adjusted for BMI, waist circumference and HbA1c.

CI confident interval, *OR* odds ratio, *NAFLD* non-alcoholic fatty liver disease, *NASH* non-alcoholic steatohepatitis
